# Insecticide Resistance in *Aedes aegypti* from the National Capital Region of the Philippines

**DOI:** 10.3390/insects15100782

**Published:** 2024-10-09

**Authors:** Richard Paul B. Malijan, Jason R. Angeles, Ariza Minelle A. Apilado, Mary Ann T. Ammugauan, Ferdinand V. Salazar

**Affiliations:** Department of Medical Entomology, Research Institute for Tropical Medicine, 9002 Research Drive, Filinvest Corporate City, Alabang, Muntinlupa City 1781, Philippines; richard.malijan@ritm.gov.ph (R.P.B.M.); jsn.rvc.angeles@gmail.com (J.R.A.); ariza.apilado@ritm.gov.ph (A.M.A.A.); mary.ammugauan@ritm.gov.ph (M.A.T.A.)

**Keywords:** *Aedes aegypti*, insecticide resistance, Philippines, National Capital Region

## Abstract

**Simple Summary:**

The National Capital Region (NCR), Philippines, reports one of the country’s highest dengue incidences. Apart from being populous and the center of economic activity, the local government authorities of this region have undertaken significant vector control efforts devoted to dengue. The use of insecticides to reduce mosquito vector density remains the handiest control method. This scenario necessitated the documentation of the resistance levels, particularly of the most important vector, *Aedes aegypti*. An insect is said to be resistant when the known effective dose of an insecticide can no longer sufficiently kill the same insect population. This study showed that *Ae. aegypti* population from cities in NCR had developed resistance to commonly used pyrethroids (permethrin, etofenprox) and organochlorine (DDT). Highly localized variations of resistance and susceptibility within cities at NCR were recorded against deltamethrin, cyfluthrin, and lambda-cyhalothrin. This finding should alert public health authorities to consider modifying the existing vector management package for greater control efficacy.

**Abstract:**

Human arboviral diseases such as dengue, chikungunya, and Zika can be transmitted by the mosquito *Aedes aegypti*. The insecticide-based vector control strategy is critical in reducing transmission of these *Aedes*-borne diseases but is threatened mainly by the emergence of insecticide resistance. Adult *Ae. aegypti* from the National Capital Region (NCR), Philippines, were subjected to bioassays to determine their susceptibility to diagnostic doses of pyrethroid, organochlorine, and organophosphate insecticides following the standard World Health Organization insecticide susceptibility test. This study reports the detection of insecticide resistance to pyrethroids and organochlorine in *Ae. aegypti* from the Philippines for the first time. Most of the *Ae. aegypti* populations from NCR exhibited phenotypic resistance to permethrin, etofenprox, and DDT. Varying resistance levels to deltamethrin, cyfluthrin, and lambda-cyhalothrin were observed in the different mosquito populations, while all populations tested to malathion were susceptible to this organophosphate. This finding should alert public health authorities to consider modifying the existing vector management package for greater control efficacy. Best practices proven to prevent or delay the development of insecticide resistance, such as insecticide rotation, should also be implemented, while alternative chemicals with a different mode of action should be explored to ensure the continuing efficacy of program interventions.

## 1. Introduction

The public health implications of dengue infection in the Philippines have been significant since its first recorded outbreak [1]. The high numbers of reported cases in previous years showed that it has been causing damaging effects on human lives. In 2019, the Philippine Department of Health (DOH) declared a National Dengue Epidemic due to the significantly higher cases than in previous years [2]. However, the impact of community lockdowns to contain the COVID-19 pandemic has dropped national dengue figures significantly [3,4]. This situation supports earlier claims that routine house-to-house human movements aid the spread of dengue in localities [5], much so that human travel on both local and global scales represents a significant global health risk, particularly in areas with changing climatic suitability for the mosquito vector [6].

Human arboviral diseases such as dengue, chikungunya, and Zika remain a global public threat even in the advent of the worldwide coronavirus pandemic [7,8]. *Aedes aegypti* transmits these diseases, a highly adaptive species that thrives in urban and suburban areas. There is no specific prophylactic treatment nor readily accessible vaccine for *Aedes*-borne diseases to date. The future of vaccines against dengue remains under litigation due to perceived side effects after the initial mass administration, which resulted in citizens’ hesitancy [9]. Thus, a need remains to develop and implement vector control measures to combat such diseases.

Integrated vector control in the Philippines includes the combination of legislation, community participation, information education campaigns, environmental management, and mosquito control. Health offices nationwide follow the DOH-issued guidelines on mosquito control which includes safe handling and the correct application of larvicides (e.g., temephos and fenthion) and adulticides for targeted indoor/outdoor residual spraying and space spraying during outbreaks (e.g., permethrin, deltamethrin, cyfluthrin, pirimiphos-methyl, and malathion), implementation of programs like *Aksyon Barangay Kontra Dengue (ABKD)*, search and destroy of mosquito breeding sites or the “4 o’clock habit”, 4S strategy [10,11,12,13,14,15]. Insecticide use has been the cornerstone of *Aedes* vector control in the country. Personal communication with the Regional of Entomologist of NCR (Dominic A. Sotto, RN, MMHeA, 14 August 2013) listed the following active ingredients (AI) of insecticides that were downloaded to local health facilities for mosquito control: permethrin, deltamethrin, pyriproxifen, temephos, fenthion, alpha-cypermethrin, and *Bacillus thuringiensis israelensis* or Bti). The use of safe and efficacious insecticides against the adult and larval populations of mosquito vectors is one of the most effective ways to interrupt the transmission of mosquito-borne diseases rapidly [16]. The high efficacy in regulating the mosquito populations with relatively rapid action of insecticide application makes it the most extensively practiced control of *Ae. aegypti* [7]. Space-spray techniques such as thermal fogging and ultra-low volume (ULV) spraying are utilized to control *Ae. aegypti* adults result in immediate kill effects that drastically lower mosquito populations after declaring an epidemic [17,18]. However, with greater coverage and regular use for mosquito control, there is a higher potential risk for vector mosquitoes to develop some form of resistance, such as, but not limited to, target site resistance, metabolic resistance, penetration resistance, and behavioral resistance. The steep increase in insecticide-based intervention means increased exposure to potential insecticide selection pressure on the vector. With the regular use of insecticides, it is important to monitor the development of resistance patterns at the earliest possible stage to assess their likely impact on control operations [19]. Currently, *Ae. aegypti* and *Ae. albopictus* has already been reported to have developed resistance to all types of insecticides [20]. Resistance development has generally been due to overuse, non-judicious application, and insecticide usage for other purposes that come into contact with the two vector species. Cross-resistance and multiple resistance have been equally documented, as there were compounds not used before, but the vectors no longer exhibit susceptibility to them. However, there are no recent studies on larval or adult *Aedes* insecticide resistance in the Philippines [21]. Therefore, comprehensive and regular susceptibility testing is required to form an essential adjunct to any insecticide-based control operation. An insecticide must be selected to which the mosquito vectors are susceptible to ensure the insecticide-based interventions are cost-effective.

This study investigated the insecticide susceptibility or resistance status of the primary dengue vector, *Ae. aegypti*, from the National Capital Region (NCR) based on the standard World Health Organization (WHO) contact test using diagnostic doses. Determining the resistance in the mosquito vectors will provide essential information for health planners and disease control specialists in selecting alternative compounds and modifying vector control strategies currently implemented. Resistance data should also propel further studies on the mechanisms of resistance.

## 2. Materials and Methods

### 2.1. Mosquito Collections

**Figure 1:**
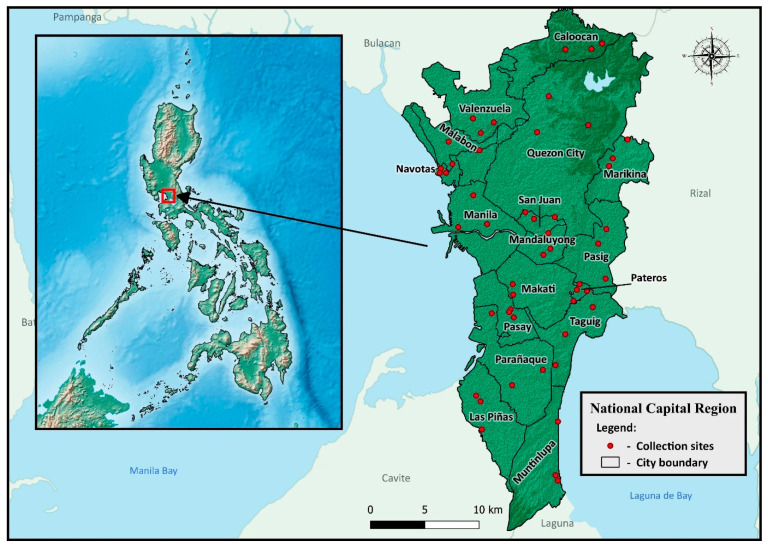
Location of mosquito collection sites in the cities/municipalities of the National Capital Region.

Collection of mosquitoes was performed in 16 cities and 1 municipality of the National Capital Region (NCR), also known as Metropolitan Manila, located in the southwestern portion of Luzon (Figure 1). NCR is the smallest administrative region (620 km^2^) but is the second most populous region, with approximately 13.5 million inhabitants [22]. In each city/municipality, three barangays (brgy.) (*n* = 51 barangays) were selected for entomological surveys (Table S1) based on the current and previously recorded dengue cases and the City Health Offices’ recommendations. Barangay, the native Filipino term for a village, is the smallest administrative division in the country. It is under a component city or municipality of a province belonging to a geographical region.

One-time ovitrapping of mosquitoes was carried out between September 2013 and June 2015. The ovitrap consists of 300 mL plastic pots, two-thirds filled with water, and a wooden stick (paddles) made of high-density fiberboard wrapped with filter paper was added as an oviposition medium. The ovitraps were installed indoors and outdoors in 30 houses per barangay. The wooden paddles and water, which may be present with immature mosquitoes, were collected after one week and placed in labeled plastic bags.

### 2.2. Mosquito Rearing

All collected immature stages from the ovitraps were reared to adults in the laboratory for morphological identification using available dichotomous keys [23]. *Aedes* collections were segregated according to species and collection site and reared in the insectary at 26 ± 2 °C and 60 ± 10% relative humidity. Due to limited *Ae. albopictus* collected, only *Ae. aegypti* were grown continuously for testing. The larvae were fed with ground fish flakes (Tetrafin ^®^) and Brewer’s yeast, while adults were provided with 10% sugar solution ad libitum. Female mosquitoes were given blood meals using white mice to induce egg-laying. The rearing of the mosquitoes continued until the F_1_ and F_2_ generations to produce enough adult females for susceptibility testing.

### 2.3. Insecticide Susceptibility Tests

Tube bioassays were carried out using standard insecticide-treated papers following the 2013 WHO guidelines available at the time of testing [24,25,26]. Insecticide- and oil-impregnated control papers were sourced from the Vector Control Research Unit, Universiti Sains Malaysia, a WHO Collaborating Center. The following diagnostic concentrations of insecticides were used: pyrethroid (0.75% permethrin, 0.05% deltamethrin, 0.15% cyfluthrin, 0.05% lambda-cyhalothrin, 0.05% etofenprox), organochlorine (4% DDT), and organophosphate (5% malathion). Diagnostic doses recommended for *Aedes* in the WHO adult bioassays have not yet been defined for some insecticides; thus, *Anopheles* mosquito’s listed diagnostic doses were used in this study. These doses are higher than *Aedes’* for the few insecticides defined in 1992 [26] and were also used in similar published studies [27,28,29,30]. Due to the failure to find a fully susceptible strain from local collections, the *Ae. aegypti* Bora-Bora strain sourced from Singapore, Melaka, and Selangor strains from Malaysia were used as references. A susceptibility test was performed per insecticide for each batch of impregnated paper from University Sains Malaysia. Reference strains (Bora-Bora, Melaka, and Selangor) of *Ae. aegypti* showed full susceptibility to deltamethrin (100%), etofenprox (99%), permethrin (100%), malathion (100%), and lambda-cyhalothrin (100%) with control mortalities ranging from 0 to 3.33%.

For each insecticide, 5 batches of 20 non-blood-fed *Ae. aegypti* females (5–7 days old) were introduced into holding tubes lined with untreated paper for 60 min for the exposed group, while 3 batches of 20 mosquitoes were provided as the control group. Mosquitoes were transferred into exposure tubes lined with insecticide-treated or control paper for 1 h. This study modified the WHO procedure (i.e., recording of knockdown every 5 min interval) to develop a sensitive detection method for monitoring resistance trends [31,32]. After exposure, mosquitoes were transferred back to the holding tubes and had *ad libitum* access to the sugar solution. Mortality was determined 24 h post-exposure in each replicate.

### 2.4. Data Analysis

The mortality rate for each insecticide was calculated by adding the number of dead mosquitoes on all five exposure replicates and expressing it as a percentage of the total number of exposed mosquitoes. A similar calculation was made for the control mortality. When control mortality was between 5 and 20%, mortality was corrected using Abbott’s formula [33], with negative values rounded to zero. Following the WHO guidelines [25], mosquito populations were considered susceptible if the percentage of mortality ranged from 98 to 100% at 24 h post-exposure. Mosquito mortality between 90 and 97% was defined as incipient or developing resistance but would require follow-up testing to confirm resistance. If mortality is less than 90%, phenotypic resistance is confirmed if at least 100 mosquitoes are tested.

## 3. Results

Immature stages of *Ae. aegypti*, *Ae. albopictus*, and *Cx. quinquefasciatus* were collected from the ovitraps placed in households in 51 barangays in the NCR. However, bioassays were performed only for *Ae. aegypti* from 32 barangays under the 13 cities/municipalities due to the insufficient test specimens for the other two species. Mortality and knockdown (KD) rates for each mosquito strain are reported, including only those tests with at least 100 mosquitoes in all replicates in the final analysis.

### 3.1. Mortality Rates

**Figure 2:**
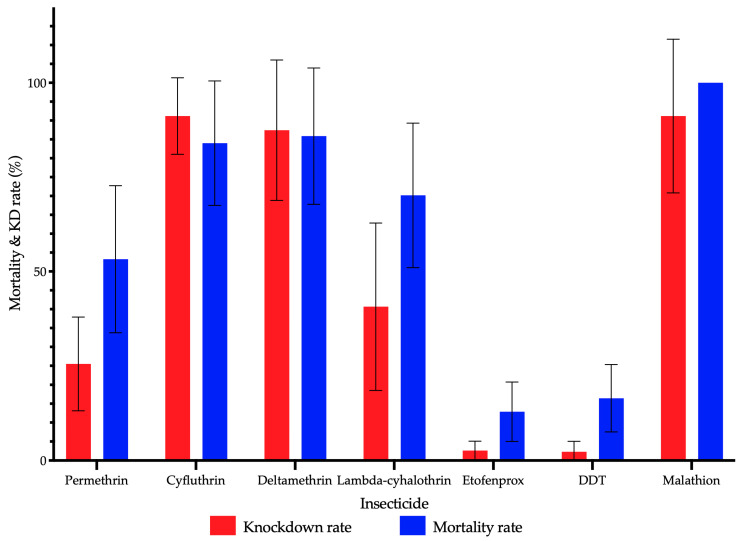
Knockdown (red bars) and mortality (blue bars) rates determined following the WHO susceptibility test procedure. Error bars represent 95% confidence intervals.

**Figure 3:**
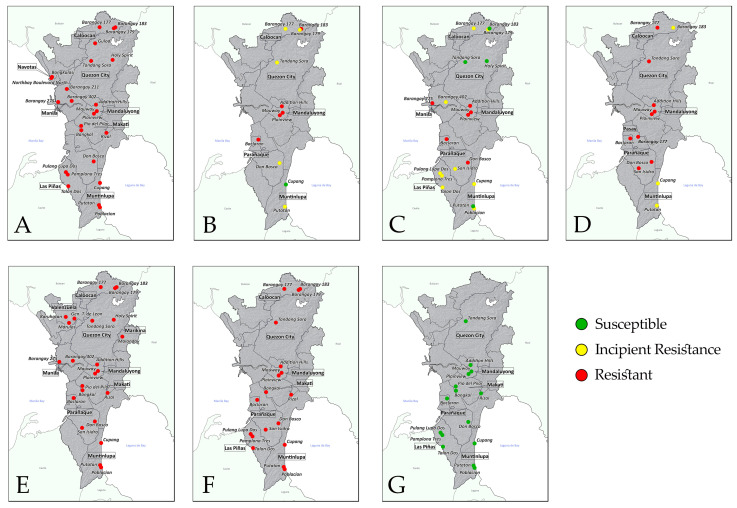
Insecticide resistance of *Ae. aegypti* across barangays in different cities within NCR from September 2013 and June 2015: (**A**) permethrin, (**B**) cyfluthrin, (**C**) deltamethrin, (**D**) lamba-cyhalothrin, (**E**) etofenprox, (**F**) DDT, and (**G**) malathion.

Mosquito populations from the different barangays showed varying mortality rates based on the WHO susceptibility tests conducted against different insecticides (Figure 2). Results showed varying levels of insecticide resistance of *Ae. aegypti* across barangays in different cities within NCR (Figure 3). Resistance to permethrin and etofenprox was detected in all tested *Ae. aegypti* populations from 24 and 25 barangays in the NCR, respectively, with mortality rates ranging from 1.00% to 86.91% (mean = 52.28%, 95% confidence interval = 40.53–64.02%) for permethrin and 0.00% to 40.00% (mean = 14.34%, 95% CI = 8.68–20.00%) for etofenprox. Exposure to deltamethrin yielded mortality rates ranging from 41.00% to 100.00% (mean = 85.11%, 95% CI = 74.97–95.24%), indicating varying resistance levels in the *Ae. aegypti* populations. Over half of the populations (10 out of 18) showed incipient resistance to deltamethrin, while 28% (5/18) had confirmed phenotypic resistance. The remaining 17% (3/18) were still deltamethrin-susceptible, namely, barangays 183 (Manila), Putatan (Muntinlupa City), and Tandang Sora (Quezon City) showed susceptibility to deltamethrin. Varying resistance levels to cyfluthrin were also detected in tested populations with mortality rates from 50.00% to 100.00% (mean = 82.17%, 95% CI = 70.36–93.98%); 45% (5/11) with confirmed resistance, another 45% (5/11) showing incipient resistance, and only the *Ae. aegypti* population from Brgy. Cupang (Muntinlupa City) showing full susceptibility. Exposure to lambda-cyhalothrin rendered mortality rates ranging from 16.00% to 93.00% (mean = 69.35%, 95% CI = 54.83–83.87%), showing confirmed resistance in 75% of the tested populations (9/12) while the remaining 25% (3/12) with incipient resistance. Phenotypic resistance to DDT was observed in all (19/19) tested populations, with test mortality ranging from 2.00% to 47.00% (mean = 15.44%, 95% CI = 9.45–21.43%). In contrast, all (13/13) *Ae. aegypti* populations tested against malathion were susceptible to the insecticide, with a 100% test mortality rate in all tests.

### 3.2. Knockdown Rates

Overall knockdown and mortality rates for the NCR mosquito population are reported in Figure 2. The evolution of the KD rate over a 60-minute observation period is shown in Figure S1. The confidence intervals (CI) were determined for KD and mortality. Though mortality and KD seem similar and CI (95%) overlap for deltamethrin, their values are independent, meaning independent observations (KD and mortality) at 60 min and 24 h, respectively. Both values show the action of insecticides in the mosquitoes, meaning if the KD value is higher than mortality, mosquitoes could recover from the insecticide to which they were exposed. Overall, the mean KD rate for cyfluthrin (mean = 90.73%, 95% CI = 82.77–98.68%) and deltamethrin (mean = 86.28%, 95% CI = 75.98–96.57%) is higher compared to mortality (mean = 82.17%, 95% CI = 70.36–93.98% and mean = 85.11%, 95% CI = 75.98–96.57%, respectively), showing recovery in the mosquito population during the 24 h recovery period. In contrast, higher mortality rates than the KD rate were observed for permethrin (mean = 26.00%, 95% CI = 16.96–35.04), lambda-cyhalothrin (mean = 42.08%, 95% CI = 26.39–57.77%), etofenprox (mean = 2.72%, 95% CI 1.17–4.27%), DDT (mean = 2.28, 95% CI = 0.36–4.20%), and malathion (mean = 95.69, 95% CI = 89.48–101.91%).

## 4. Discussion

Here, we provide evidence of the resistance status of *Ae. aegypti* populations from NCR, Philippines, to insecticides used to control adult mosquitoes. *Aedes aegypti* populations from this region exhibited varying susceptibility/resistance levels to pyrethroid insecticides, confirmed resistance to DDT, and susceptibility to malathion. To the authors’ knowledge, this is the latest report on a broader scale of the insecticide resistance of *Ae. aegypti* populations from the Philippines after the insecticide efficacy studies in Cebu and Pampanga several decades ago [34,35].

There had been limited studies on insecticide resistance in mosquito vectors from the Philippines; most were focused on malaria vectors [31,32,34,36,37,38]. Early studies reported that *Ae. aegypti* from Clark Air Base in Pampanga is susceptible to DDT and dieldrin [34]. In contrast, the secondary dengue vector *Ae. albopictus* showed resistance to BHC/cyclodienes [37]. From 2005 to 2006, a thesis study detected resistance in *Ae. aegypti* populations from Manila to permethrin, cyfluthrin, deltamethrin, and DDT but were still susceptible to malathion and temephos [39]. In the same study, populations from Cabuyao, Laguna exhibited varying resistance/susceptibility to the said insecticides.

Available guidelines on adulticide application of malathion, fenitrothion, or permethrin using ULV mist blowers during dengue epidemics can be traced back to 1991 [40,41]. From 1997 to 2003, the local government units invariably used permethrin, deltamethrin, cyfluthrin, and organophosphates such as pirimiphos-methyl and malathion. From 2004 onwards, other insecticides registered with the Fertilizer and Pesticide Authority were used for space spraying, while permethrin-treated curtains were included as an additional adult control measure [35]. The use of insecticides inevitably induces selection pressures on the target vectors, particularly in the primary vector of dengue *Ae. aegypti*.

Pyrethroids were heavily used to control dengue vectors due to their immediate result, high mortality effect, low residual efficacy, and low mammalian toxicity [42,43,44]. Based on the reports of the Philippine regional health offices, chemical control activities, such as thermal fogging, space spraying, and insecticide-treated curtains, lean towards the use of pyrethroids. With the high dependence, frequency, and usage of pyrethroid-based insecticides, higher selection pressure on the insecticides could have increased mosquito resistance levels [45,46]. Efficacy studies on mosquito coils observed cross-resistance between pyrethroid insecticides (d-allethrin and transfluthrin, d-allethrin, and metofluthrin, and transfluthrin and metofluthrin) [47]. Residents rely on mosquito coils aside from bed nets and electric fans as preventive measures [48]. In addition, cheaper and broad-spectrum insecticides are also easily accessible in the market, which may contribute to developing insecticide resistance. In 2015, the Food and Drug Administration (FDA) advised the public not to buy several unregistered aerosol cypermethrin-containing insecticides since these are not FDA-compliant in efficacy and safety [49]. Alternatively, cone bioassay tests on permethrin-treated nets (e.g., Olyset net ^®^) rolled out to public elementary schools in Cagayan de Oro, a province in the southern Philippines, showed probable resistance [50]. 

High resistance to etofenprox was detected in all tested populations using the recommended diagnostic dose for *Aedes*, with the highest test mortality being only 40%. On the other hand, some level of resistance to cyfluthrin was detected in *Ae. aegypti* populations except those from Brgy. Cupang (Muntinlupa, Philippines). Similarly, cyfluthrin resistance was also observed in *Ae. aegypti* from Jakarta, Indonesia [51]. Etofenprox is also known as a pseudo pyrethroid because it is closely related to pyrethroids (types I and II). Similarly, type I (permethrin) and type II (deltamethrin) pyrethroids are closely comparable with etofenprox in terms of the P450 depleting mechanism [52]. It was discovered in the P450 depletion research that vector population, particularly *Ae. aegypti* may become more tolerant to etofenprox metabolic resistance mechanisms (such as P450). Metabolic resistance mechanisms generally act on all classes of insecticides, therefore, the possibility of cross-resistance to less used compounds. More than the high and direct public health use of etofenprox against vectors, it has also been widely used to control a range of fruit and vegetable pests, even in rice fields [53,54]. Further, etofenprox is also used as an ingredient in flea medication for cats and dogs, giving a very high possibility of *Aedes* mosquitoes [54].

The *Anopheles* diagnostic doses were used for permethrin, deltamethrin, and lambda-cyhalothrin since no defined diagnostic doses for *Aedes* were recommended when this study was conducted. Despite the higher *Anopheles* diagnostic doses, resistance was observed to type I (permethrin) and type II (deltamethrin and lambda-cyhalothrin) pyrethroids. Similar findings were observed in *Ae. aegypti* populations from neighboring Southeast Asian countries, particularly the observed resistance to permethrin [7,21,51,55,56,57,58]. All tested populations also showed some level of resistance to lambda-cyhalothrin; most tested populations had confirmed phenotypic resistance, while the remaining populations showed incipient resistance, similar to observations in Indonesia [51]. Varying levels of resistance to deltamethrin were detected in the different *Ae. aegypti* populations, with the majority showing tolerance to deltamethrin. Compared with *Ae. aegypti* from Malaysia, all tested populations were already resistant to deltamethrin, with test mortality ranging from 0 to 82% [58]. In contrast, *Ae. aegypti* populations from Laos are mostly susceptible to the insecticide [59].

DDT has long been banned in the country [40,60], yet insecticide resistance has been observed in mosquito populations. Pyrethroids and organochlorines have the same mode of action that interrupts nerve impulses in nerve axons, causing hyperexcitation and tremors followed by paralysis and blocking of nerves in mosquitoes [7,61]. Resistance of the *Ae. aegypti* populations to pyrethroids may have conferred the populations’ cross-resistance to DDT, and vice versa. However, further studies are needed to establish the possible cross-resistance to the two insecticide classes. Mutations in resistance against permethrin and deltamethrin have already been reported to be linked to DDT resistance, showing that there can be cases of cross-resistance among the two insecticide groups [62,63]. DDT’s persistence in the environment is also considered one of the causes of resistance [64,65].

Organophosphates have been heavily used for dengue vector control and agriculture, leading to intense selection pressure and highly resistant mosquitoes in other countries [66]. In the Philippines, organophosphates such as malathion are often used against agricultural pests but also against dengue vectors by the different local government units [40]. The high susceptibility of *Ae. aegypti* to malathion may be attributed to minimal use of the compound for dengue vector control. The community has poorly accepted malathion due to its repulsive odor. However, regular resistance monitoring for this insecticide is needed as resistance to malathion has already been reported in neighboring countries [7,51,67]. A more detailed retrospective study is recommended to determine how these compounds were used for vector control that would impact the judicious use and environmental safety. This study should, therefore, influence the current DOH policy and the advocacy of the National *Aedes*-borne Viral Disease Prevention and Control Program.

Promoting the responsible use of pesticides in the agricultural and public health sectors is crucial to managing insecticide resistance. To delay the development of resistance, the WHO recommendations for managing insecticide resistance include (1) the judicious use of insecticides, (2) the use of different AI between complementary interventions (i.e., different AI for outdoor misting with the AI for targeted indoor residual sprays, (3) mosaic insecticide application or the use of different compounds in neighboring areas, and (4) insecticide rotation with compounds of a different mode of action with the documented resistance. The data suggest alternative use of non-pyrethroids such as organophosphates or other compounds with different modes of action. The use of insecticide AI with synergists (piperonyl butoxide) or combination actives with additives that enhance the killing effect can also be resorted to. Public health authorities should check if the insecticides being considered have been registered with the Philippines FDA or similar agencies in other countries. More recent and continuous insecticide resistance monitoring and further vector behavior studies must be performed. Characterizing resistance mechanisms will provide critical information in choosing alternative compounds, application design and frequencies, and more ways to prevent resistance and effectively use chemicals for vector control. Furthermore, in the health management sector, it is imperative to enhance the provision of resources for monitoring insecticide resistance, such as to enhance capacity-building activities, increase the budget allocation, and, most importantly, develop a national strategic plan for vector surveillance and control with systematic coordination with local health units, private sector, and other related stakeholders.

Current resistance levels are essential in developing an effective vector management program. Therefore, regular insecticide resistance monitoring must be operationalized in the NCR and the Philippine islands. Further studies on the molecular characterization of the insecticide resistance mechanism will provide alternatives for the vector control program toward more effective implementation of an insecticide resistance management plan in the context of integrated vector management. The data presented here were collected in 2013–2015. This data set is the only available information for resistance in *Ae. aegypti* in the Philippines and, therefore, highlights the need for an updated assessment of the status of vector resistance to compounds used in vector control.

## 5. Conclusions

Results from this study focused on phenotypic resistance in *Ae. aegypti* populations in NCR, Philippines. Nonetheless, this study provides pertinent information on *Ae. aegypti*’s general susceptibility or resistance to insecticides, serves as baseline data for future studies on insecticide resistance in dengue vectors, and guides public health authorities and program managers. Moreover, this report should signal an update on the vector control program’s current insecticide resistance management plan. Further studies on the characterization of resistance mechanisms will help provide the impetus for developing newer compounds and continued studies on mosquito resistance’s molecular/genetic basis and the possibility of gene manipulation to maintain susceptibility in vector populations.

There is a need to assess the operational impact of pyrethroid use in controlling adult mosquitoes. Using compounds with a different mode of action from pyrethroids is highly recommended. Since the current formulation of malathion renders it unacceptable to the community, reformulation of the product by the manufacturers may help increase acceptance and use in dengue vector control. In the meantime, the Dengue Control Program’s 5S advocacy is highly encouraged for disease prevention and control: (1) search and destroy mosquito breeding sites, (2) secure self-protection, (3) seek early consultation, (4) support fogging/spraying only in hotspot areas where an increase in cases is registered for two consecutive weeks to prevent an impending outbreak, and (5) sustain hydration.

## Data Availability

All relevant data are within the article and its Supplementary Materials Files. Raw data are available from the corresponding author upon reasonable request through the Research Institute for Tropical Medicine Director.

## Supplementary Materials

The following are available online at https://www.mdpi.com/article/10.3390/insects15100782/s1, Table S1: GPS coordinates of mosquito collection sites in the National Capital Region, Philippines; Figure S1: Changes in the knockdown rate during insecticide exposure recorded every five minutes during the exposure period to insecticide.
