# Supplementary material for: Insecticide Resistance in Aedes aegypti from the National Capital Region of the Philippines
Source: Insects. 2024 Oct 9;15(10):782. doi: 10.3390/insects15100782 (PMC11508968; doi:10.3390/insects15100782)
Supplement: Supplementary file 1 [file insects-15-00782-s001.zip › Supp Material Figure. Changes in KD Rate.pdf]

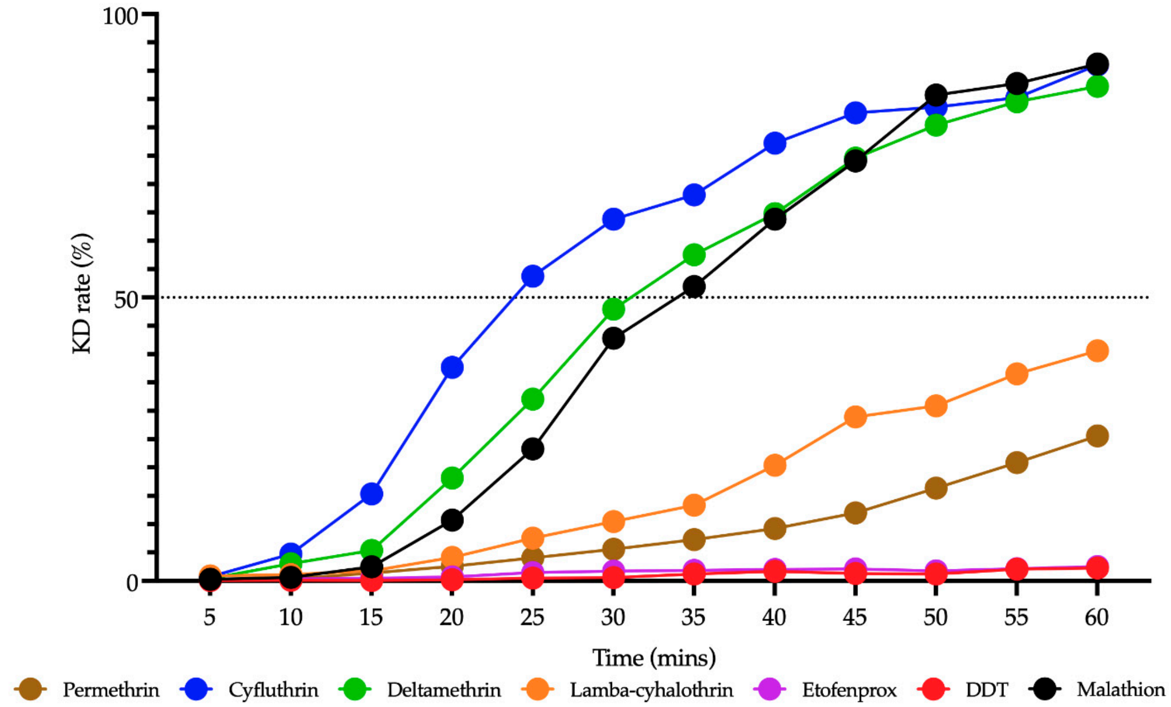

Figure S1#. Changes in the knockdown rate during insecticide exposure recorded every five minutes during the exposure period to insecticide. The line indicates a 50% KD rate.
