# Supplementary material for: Insecticide Resistance in Aedes aegypti from the National Capital Region of the Philippines
Source: Insects. 2024 Oct 9;15(10):782. doi: 10.3390/insects15100782 (PMC11508968; doi:10.3390/insects15100782)
Supplement: Supplementary file 1 [file insects-15-00782-s001.zip › Supp Material Table S1. GPS coordinates of mosquito collection sites in the National Capital Region, Philippines.pdf]

Table S1. GPS coordinates of mosquito collection sites in the National Capital Region, Philippines

City/Municipality	Barangay	Latitude (N)	Longitude (E)
Manila	Barangay 211	14.62597	120.98231
	Barangay 275	14.60011	120.96463
	Barangay 402	14.60223	120.99233
Mandaluyong	Addition Hills	14.59476	121.04212
	Mauway	14.58190	121.04386
	Plainview	14.57685	121.03797
Marikina	Malanday	14.65019	121.09383
	Nangka	14.67336	121.10869
	Tumana	14.65639	121.09712
Pasig	Manggahan	14.59810	121.09162
	Pinagbuhatan	14.55717	121.09093
	Rosario	14.58607	121.08468
Quezon City	Gulod	14.71677	121.04043
	Holy Spirit	14.68383	121.07628
	Tandang Sora	14.68177	121.03223
San Juan	Corazon de Jesus	14.60659	121.03145
	Salapan	14.61301	121.02480
	West Crame	14.60747	121.05089
Caloocan	Barangay 177	14.74877	121.04957
	Barangay 179	14.74664	121.07886
	Barangay 183	14.74856	121.08196
Malabon	Catmon	14.67023	120.96010
	Longos	14.65172	120.96031
	Potrero	14.66316	120.98342
Navotas	Bangkulasi	14.65003	120.95207
	Northbay Boulevard North	14.64773	120.95065
	Northbay Boulevard South	14.64671	120.95487
Valenzuela	Gen. T. De Leon	14.68616	120.99555
	Karuhatan	14.68945	120.97783
	Marulas	14.67744	120.98450
Las Piñas	Pamplona Tres	14.45571	120.98427

City/Municipality	Barangay	Latitude (N)	Longitude (E)
	Pulang Lupa Dos	14.46056	120.98054
	Talon Dos	14.43262	120.98550
Makati	Bangkal	14.54397	121.01214
	Pio del Pilar	14.55258	121.01175
	Rizal	14.53873	121.06356
Muntinlupa	Cupang	14.43909	121.05035
	Poblacion	14.39052	121.05024
	Putatan	14.39494	121.04831
Paranaque	Baclaran	14.52849	120.99387
	Don Bosco	14.48186	121.03747
	San Isidro	14.46922	121.01123
Pasay	Barangay 177	14.53177	121.00995
	Barangay 183	14.52510	121.01266
	Barangay 184	14.52954	121.00872
Pateros	Sto. Rosario Kanluran	14.55263	121.06878
	San Pedro	14.54697	121.06639
	Santa Ana	14.52795	121.07690
Taguig	Calzada	14.53366	121.08003
	Central Signal Village	14.51136	121.05654
	North Daang Hari	14.48581	121.04827
